# Genome and transcriptome profiling of fibrolamellar hepatocellular carcinoma demonstrates p53 and IGF2BP1 dysregulation

**DOI:** 10.1371/journal.pone.0176562

**Published:** 2017-05-09

**Authors:** Eric C. Sorenson, Raya Khanin, Zubin M. Bamboat, Michael J. Cavnar, Teresa S. Kim, Eran Sadot, Shan Zeng, Jonathan B. Greer, Adrian M. Seifert, Noah A. Cohen, Megan H. Crawley, Benjamin L. Green, David S. Klimstra, Ronald P. DeMatteo

**Affiliations:** 1 Department of Surgery, Memorial Sloan Kettering Cancer Center, New York, New York, United States of America; 2 Department of Computational Biology and Bioinformatics Core, Memorial Sloan Kettering Cancer Center, New York, New York, United States of America; 3 Department of Pathology, Memorial Sloan Kettering Cancer Center, New York, New York, United States of America; Beijing Cancer Hospital, CHINA

## Abstract

Fibrolamellar hepatocellular carcinoma (FL-HCC) is a rare variant of HCC that most frequently affects young adults. Because of its rarity and an absence of preclinical models, our understanding of FL-HCC is limited. Our objective was to analyze chromosomal alterations and dysregulated gene expression in tumor specimens collected at a single center during two decades of experience with FL-HCC. We analyzed 38 specimens from 26 patients by array comparative genomic hybridiziation (aCGH) and 35 specimens from 15 patients by transcriptome sequencing (RNA-seq). All tumor specimens exhibited genomic instability, with a higher frequency of genomic amplifications or deletions in metastatic tumors. The regions encoding 71 microRNAs (miRs) were deleted in at least 25% of tumor specimens. Five of these recurrently deleted miRs targeted the insulin-like growth factor 2 mRNA-binding protein 1 (*IGF2BP1*) gene product, and a correlating 100-fold upregulation of *IGF2BP1* mRNA was seen in tumor specimens. Transcriptome analysis demonstrated intrapatient tumor similarity, independent of recurrence site or time. The p53 tumor suppressor pathway was downregulated as demonstrated by both aCGH and RNA-seq analysis. Notch, EGFR, NRAS, and RB1 pathways were also significantly dysregulated in tumors compared with normal liver tissue. The findings illuminate the genomic and transcriptomic landscape of this rare disease and provide insight into dysregulated oncogenic pathways and potential therapeutic targets in FL-HCC.

## Introduction

Fibrolamellar hepatocellular carcinoma (FL-HCC) is a rare variant of HCC that most commonly occurs in young adults without a history of underlying liver disease [1, 2]. FL-HCC accounts for less than 1% of all primary liver malignancies and has nearly equal incidence among genders, with a mean age at diagnosis around 23–39 years [3, 4]. Clinically, FL-HCC tumors are large and well circumscribed, and patients frequently present with lymph node or distant metastasis [5]. Histologically, tumors demonstrate a distinct pattern of collagen-rich lamellar arrays of fibrous tissue surrounding nests and cords of large, polygonal hepatocyte-like cells with oncocytic features; these cells demonstrate immunohistochemical evidence of both hepatocellular and bile duct differentiation [6]. Elevated serum levels of alpha-fetoprotein are uncommon in patients with FL-HCC, but elevated serum and tumor expression of neurotensin (NTS) has been described [7, 8].

Surgical resection of FL-HCC remains the mainstay of therapy for localized disease. A number of chemotherapeutic treatments have been investigated for advanced disease but have limited efficacy [9]. FL-HCC has traditionally been considered biologically less aggressive than conventional HCC given its better overall survival [10]. However, improved prognosis may be largely secondary to a younger patient population without liver disease, a much larger fraction of whom are able to undergo potentially curative resection [11]. Unfortunately, recurrences are common and can occur many years after initial surgery [2, 4], although patients who develop recurrence may benefit from repeat hepatectomy or metastasectomy [2, 4, 10, 12].

Because of the rarity of FL-HCC and an absence of animal models or FL-HCC cell lines, relatively little of its molecular biology is understood. Reports on comparative genomic hybridization (CGH) have been limited by small numbers of patients and low-resolution CGH techniques [13–15]. Similarly, RNA expression data had been focused on individual genes in a few patients until the recent description of gene expression array data in 14 FL-HCC patients [16]. Recently, a recurrent fusion transcript was discovered in 11 patients and may be an oncogenic driver in FL-HCC [17].

In the current study, we performed high-resolution aCGH to detect copy number variations and RNA-seq on specimens from 26 and 15 FL-HCC patients, respectively, who underwent surgery at our center during the last two decades. We found that all tumors harbored chromosomal amplifications and deletions, and these alterations were more frequent in metastatic specimens (recurrent liver tumors, metastatic lymph nodes, and peritoneal metastases) compared with primary tumors. We additionally demonstrated by transcriptome analysis that metastatic tumors closely resembled the gene expression profile of the corresponding primary tumor. Furthermore, we found evidence by both aCGH and RNA-seq that p53 tumor suppressor pathway genes were significantly downregulated. Overall, Notch, EGFR, NRAS, and RB1 pathways were also dysregulated in FL-HCC.

## Patients and methods

### Patient and sample selection

Patient data and specimens were collected under approval of the Memorial Sloan Kettering Cancer Center Institutional Review Board. Tumor specimens were available from 32 patients who underwent surgery for FL-HCC between 1993 and 2012 and who provided informed consent for specimen research studies. Representative histologic slides from formalin-fixed, paraffin-embedded (FFPE) or fresh frozen (FF) tumor specimens were reviewed by a hepatobiliary pathologist (D.S.K.) to confirm the diagnosis of FL-HCC, or, in the case of normal liver tissue, the absence of tumor involvement. After pathologic confirmation, specimen processing, and quality control assays, 38 FFPE and 35 FF samples from 28 patients remained available for further analysis.

### Transcriptome sequencing

RNA was extracted from fresh frozen tissue using the Roche High Pure FFPE kit (Roche Diagnostics). After Ribogreen quantification and quality control via Agilent BioAnalyzer, 0.5 to 1 μg of total RNA underwent stranded TruSeq library preparation according to the manufacturer’s instructions (Illumina), with 6 cycles of PCR. Samples were barcoded and run on a HiSeq 2000 in a 50bp/50bp paired-end run, using the TruSeq SBS Kit v3 (Illumina). An average of 69 million paired reads were generated per sample. Ribosomal reads represented at most 0.83% of total reads, while mRNA bases accounted for an average of 61% of reads.

### DNA profiling

Genomic DNA from FFPE samples was extracted using the Agilent protocol for oligonucleotide array-based CGH for genomic DNA analysis. Human control genomic DNA (Roche) was used as the reference. DNA integrity was verified on a 1% agarose gel. 3 μg of DNA was then heat-fragmented and labeled with the ULS Labeling V3.4 kit, according to the manufacturer’s instructions (Agilent). Labeled DNA was hybridized to Agilent 1M CGH arrays for 40 hours at 60°C. After washing, the slides were scanned and images quantified using Feature Extraction 9.1 (Agilent).

### CGH data analysis

The aCGH data were first processed using a normalization procedure to correct for block, row, column or intensity effects as well as any artifact such as dependence of log-ratios on the genomic GC content. The normalized data were then segmented using the Circular Binary Segmentation algorithm [18]. The data were analyzed with the RAE algorithm designed to robustly map chromosomal alterations in tumor samples [19]. aCGH data have been submitted to the Gene Expression Ombnibus repository (GEO; http://www.ncbi.nlm.nih.gov/geo/; accession number GSE64103).

### RNA sequencing data analysis

The RNA-seq pipeline was designed to reflect the recent comprehensive evaluation of the available RNA-seq packages using the extensively characterized SEQC samples (as part of the MACQ-III consortium) and uses the negative binomial distribution to model the count data [20]. Briefly, the short reads (FASTQ files) were first mapped to the hg19 reference human genome using the TopHat2 tool [21]. After passing quality control tests with Picard tools (http://broadinstitute.github.io/picard), reads were assembled into transcripts and quantified using the htseq script [22]. Differential analysis was done using the DESeq package in R statistical language [22], and genes with a fold change of ≥2 using a false discovery rate of 0.05 were considered significant. For clustering purposes, data were normalized using the normalization procedure from the DESeq package. Human mRNA targets for miRs were generated using TargetScan [23]. RNA-seq data have been submitted to GEO (accession number GSE63018).

### RNA-seq sample clustering

Clustering of log2-normalized RNA-seq data was done using the partitioning around medoids (PAM) method (pam package) in R. Heat maps were generated using the enhanced heatmap function heatmap.2 from gplots R package. Hierarchical clustering of samples and genes was done using the Euclidian distance (hclust) function in R.

### Pathway analysis

Pathway and gene set analysis (GSA) was performed using the piano Bioconductor package [24]. The runGSA function was run with default parameters using fold changes as gene-level statistics and gene set collections from the Broad Molecular signatures database (MSigDB) [25]. Only gene-sets with adjusted p-values <0.01 are reported. An additional filtering step was applied that limited gene-sets to those in which at least half of the genes in the gene set showed fold changes of at least 50%.

### Statistical analysis

Unpaired two-tailed Student’s t tests were performed where appropriate using Prism 6.0 (Graph-Pad Software). A p-value <0.05 was considered significant.

## Results

### Genomic alterations are common in FL-HCC

**Fig 1:**
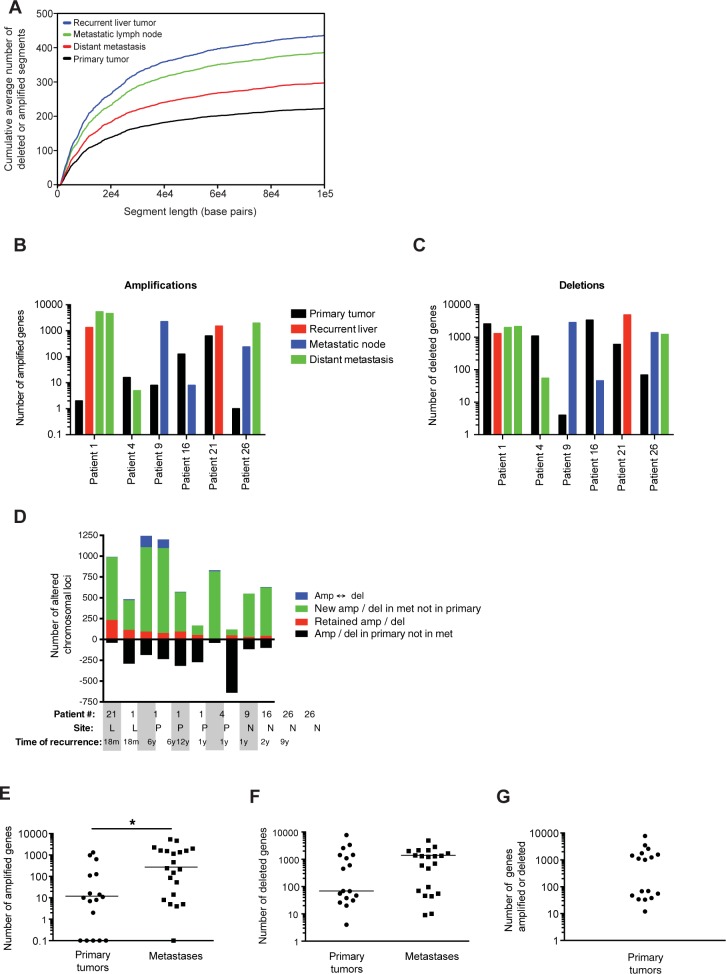
Genomic alterations are common in FL-HCC. (A) Cumulative average number of genomic segment amplifications and deletions in primary liver tumors, recurrent liver tumors, metastatic lymph nodes, and distant metastases. (B) Total number of genes amplified or deleted (C) in patients with primary and metastatic lesions. (D) Number of amplifications (amp) and deletions (del) gained, lost, or retained in metastatic tumors of the liver (L), peritoneum (P), or lymph nodes (N). Amp ↔ del refers to genes amplified in primary but deleted in metastatic tumors, or deleted in primary but amplified in metastatic tumors. Number of genes amplified or deleted in primary or metastatic tumors for all patients (E,F) and distribution of genomic alterations in primary tumors (G). Lines on dot plots represent medians. *, p<0.05.

To determine tumor genomic amplifications and deletions, we performed aCGH analysis on 38 tumor specimens from 26 patients (Table 1). All 38 samples showed evidence of genomic instability, with a mean of 405 amplified and deleted genomic loci per sample (data not shown). Recurrent liver tumors had the highest mean number of chromosomal amplifications and deletions, followed by metastatic lymph nodes and peritoneal metastases; primary tumors had the least genomic instability (Fig 1A). Similarly, in 4 of 6 patients who had paired primary and metastatic tumor specimens available, genomic amplifications were more frequent in the metastases when compared with the matched primary tumors (Fig 1B). In 3 of the 6 patients, genomic deletions were more frequent in the metastatic samples (Fig 1C). In these 6 patients, we further analyzed amplifications and deletions to determine whether metastatic specimens retained the same genomic alterations found within the primary tumor. Surprisingly, only 1 metastatic tumor specimen (from patient 21) closely resembled the primary tumor, with 234 amplifications or deletions of the 273 (86%) present in the primary tumor; however, this recurrent liver tumor also had an additional 754 new chromosomal alterations when compared with the primary tumor (Fig 1D). The remaining nine metastatic specimens shared fewer than 30% of amplifications or deletions of the corresponding primary tumor, and seven of the nine tumors had accumulated over 250 new chromosomal alterations.

**Table 1:** Clinicopathologic characteristics of 28 patients and 73 specimens.

Characteristic	n	%
Sex		
Female	15	53.6
Male	13	46.4
Age at first resection		
< 18y	9	32.1
18–29y	14	50.0
30–39y	4	14.3
> 40y	1	3.6
Stage at presentation (AJCC-7)		
Stage 2	1	3.6
Stage 3a	11	39.3
Stage 3b	1	3.6
Stage 4a	5	17.9
Stage 4b	10	35.7
Size of primary tumor		
< 5cm	1	3.6
5–10cm	11	39.3
>10cm	16	57.1
Number of operative resections		
1	10	35.7
2	13	46.4
3	3	10.7
4	1	3.6
5	1	3.6
FFPE specimens for CGH		
Primary liver tumor	17	
Recurrent liver tumor	4	
Metastatic lymph node	4	
Distant metastasis	13	
		
Fresh frozen specimens for RNASeq		
Normal liver	9	
Primary liver tumor	7	
Recurrent liver tumor	3	
Metastatic lymph node	8	
Distant metastasis	8	

Overall, the median number of genomic amplifications was more than 20-fold higher in metastatic tumors when compared with primary tumors (*p*<0.01), whereas the difference in deletions was not statistically different (*p* = 0.9) (Fig 1E and 1F). Intriguingly, the number of genes amplified or deleted in primary tumors demonstrated a bimodal distribution, with fewer than 100 amplified genes in 8 of 17 tumors and more than 1000 amplified genes in the remaining 9 samples (Fig 1G). However, there was no difference in the likelihood of recurrence, recurrence-free survival, or overall survival between the two groups (data not shown).

### p53 pathway gene amplifications and deletions are frequent in FL-HCC

**Fig 2:**
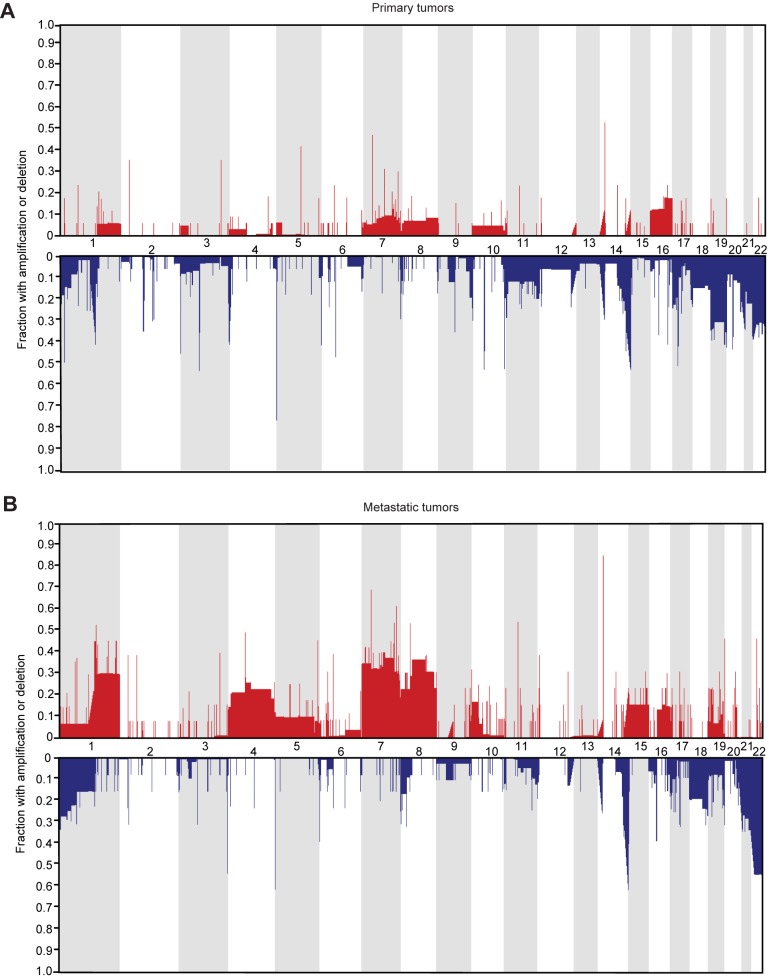
RAE analysis of genomic amplifications and deletions in FL-HCC. The frequency of genomic gain (red) or loss (blue) in primary (A) and metastatic (B) tumors is indicated on the y-axis, with relative chromosomal location on x-axis. Corresponding genetic elements are indicated in Table 2. Of note, several regions shown of high genomic gain or loss do not encompass gene coding regions and therefore do not appear in Table 2.

We performed RAE analysis[19] to map chromosomal alterations separately in primary and metastatic FL-HCC (Fig 2). The most frequently amplified and deleted chromosomal loci by RAE analysis and corresponding genes of interest are listed (Table A in S1 File). Focal genomic amplifications in 1q, 7p, 8q, and 16q were among the most commonly represented and were found in 12 to 47% of primary tumors and 16 to 81% of metastatic specimens. The most commonly deleted chromosomal loci included 10q, 6p, and 22q, and these deletions occurred in 24 to 53% of primary tumors and 14 to 57% of metastatic tumors. Pathway and gene set analysis was performed to identify significantly altered cancer-related molecular pathways (Tables B, C in S1 File). Notably, of the 28 most frequently amplified genomic elements in FL-HCC tumor specimens, 5 genes (*ANKRD11*, *IFR2BP2*, *DUSP1*, *TXNIP*, and *MCL1)* contribute to the p53 tumor suppressor pathway. Although a much larger number of genetic elements were among the most frequently deleted chromosomal loci, an additional 7 genes (*FANK1*, *PRODH*, *SLC25A1*, *MIF*, *EP300*, *GRAMD4*, and *WNT7B*) and 1 miR (*hsa-mir-33a*) with a known role in p53 pathway function were also commonly deleted in FL-HCC tumors.

### Transcriptome analysis demonstrates intrapatient tumor similarity

**Fig 3:**
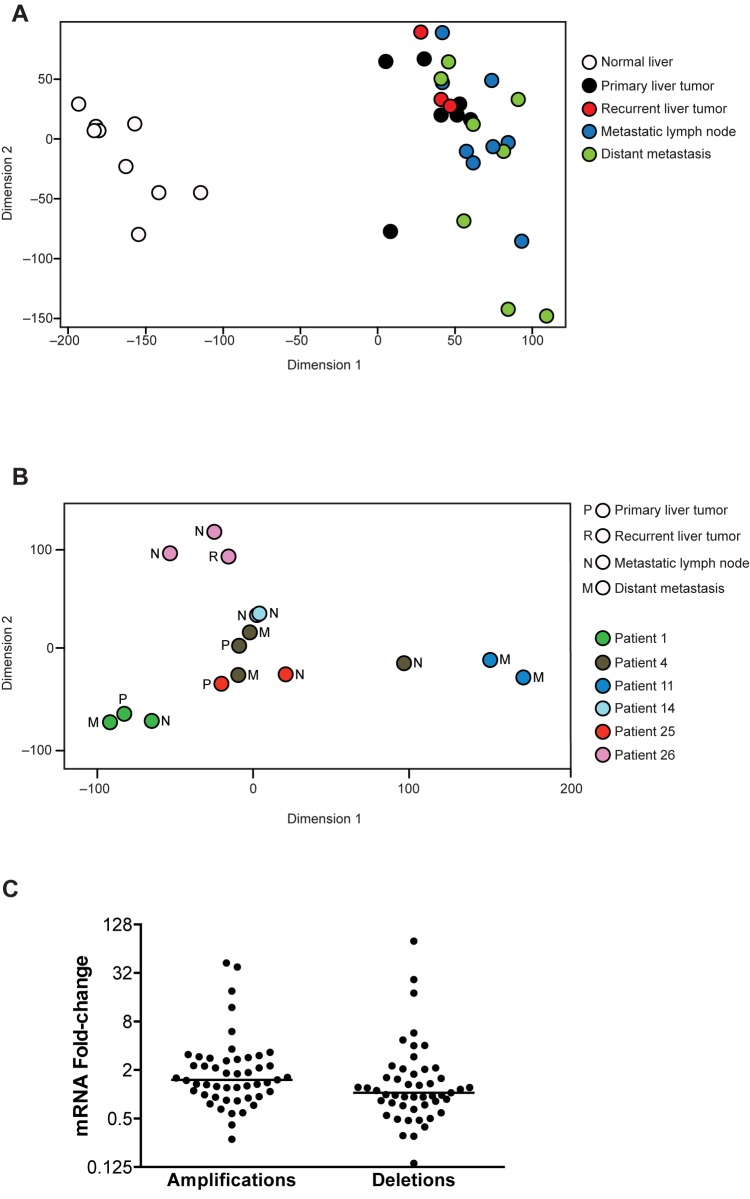
Transcriptome analysis demonstrates intrapatient tumor similarity. (A) PAM analysis of RNA sequencing data from normal liver and tumor specimens. (B) PAM analysis of RNA sequencing data from 6 patients, matched by data point color, who have multiple tumor specimens available for comparison. (C) Relative tumor mRNA expression compared with normal liver is shown for the 50 most frequently amplified and deleted genes. Lines on dot plot represent medians.

To investigate alterations in tumor gene expression, we sequenced the transcriptomes of 26 tumor specimens and 9 adjacent normal liver specimens in a total of 15 patients (Table 1). Sample clustering demonstrated that normal liver tissue clustered together and separately from primary and metastatic tumor specimens, which did not segregate (Fig 3A). Furthermore, PAM analysis focused on 6 patients with RNA from multiple tumor specimens revealed that specimens clustered by individual patient and not by specimen type (primary tumor, recurrent liver tumor, metastatic lymph node, or distant metastasis) (Fig 3B). To correlate our transcriptomic and CGH data, we analyzed differential expression of the 50 most commonly amplified and deleted genes in primary tumors. No correlation existed between amplified and overexpressed or between deleted and underexpressed genes (p = 0.99) (Fig 3C).

### p53, Notch, and EGFR signaling pathways are enriched in FL-HCC

**Fig 4:**
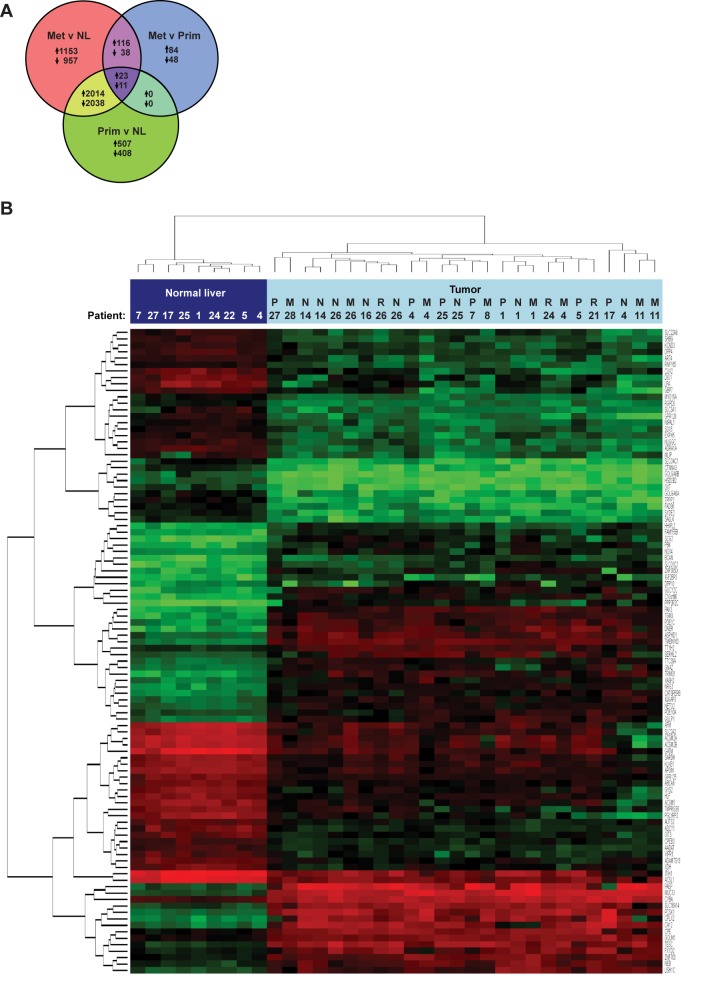
Differential gene expression reveals similarity between primary and metastatic FL-HCC. (A) Comparison of upregulated and downregulated genes in primary tumors (Prim), metastases (Met), and normal liver (NL). (B) Gene clustering diagram calculated using normalized expression values from RNA sequencing of specimens from normal liver, primary liver tumors (P), metastatic lymph nodes (N), distant metastases (M), or recurrent liver tumors (R). The 50 most significantly up-regulated and down-regulated genes between normal liver and all tumors are depicted.

A total of 7,595 genes were differentially expressed between primary tumors and normal liver, primary and metastatic tumors, and metastatic tumors and normal liver (Fig 4A). Consistent with PAM analysis, the majority (60%) of genes that were significantly up- or downregulated in primary tumors when compared with normal liver were similarly dysregulated in metastatic specimens (Fig 4A). Accordingly, genes differentially expressed between metastatic and primary specimens constituted less than 5% of the total number of dysregulated genes (Fig 4A). The most significantly up- and downregulated genes between all tumors and normal tissues are listed (Table 2; Table D in S1 File) and depicted by heat map (Fig 4B). GSA performed on fold-change data between all tumors and normal liver demonstrated dysregulation of several tumorigenesis gene sets, including angiogenesis, apoptosis, chromatin remodeling, and tumor stroma maintenance, as well as previously established gene sets specific to liver cancer; the top 40 up- and down-regulated gene sets are listed (Tables E, F in S1 File). We further filtered GSA data to focus on molecular pathways commonly dysregulated in cancer and found that the p53, Notch, EGFR, NRAS, and RB1 pathways were significantly altered in primary tumors compared with normal liver (Tables G, H in S1 File).

**Table 2:** Top 20 significantly dysregulated genes in FL-HCC tumors compared with normal liver.

Gene Symbol	adj. p-value	Fold Change	Mean Expression Normal Liver	Mean Expression Tumor
MUC13	1.3E-98	539	91	49269
PCSK1	5.4E-96	489	42	20674
PAEP	2.3E-95	736	80	58973
CPLX2	5.9E-93	661	24	15567
TMEM163	1.4E-75	187	27	4979
CA12	2.6E-67	393	43	17038
PPP2R2C	6.3E-66	435	1	382
SLC16A14	2.8E-65	118	115	13557
C10orf90	1.4E-62	125	6	768
TPPP2	9.5E-56	-64	490	8
ADRA1A	1.1E-55	-54	2766	52
USH1C	5.1E-52	51	93	4751
DNER	2.0E-51	416	9	3844
TGM3	5.4E-51	188	11	1979
SERHL2	5.7E-48	43	68	2890
NRG2	2.0E-47	45	18	804
PAK3	2.9E-47	282	9	2430
SCG2	4.3E-45	152	2	348
TESC	1.3E-44	92	177	16326
NUGGC	3.2E-44	-30	1848	61

We next analyzed our transcriptome data for the presence of fusion transcripts. To minimize the number of false positives, we applied four algorithms to detect fusion chimeras from RNA-seq data. The previously described *DNAJB1-PRKACA* fusion transcript[17] was detected by FusionCatcher and 3 additional algorithms in 26 of 26 tumors and 0 of 9 normal liver specimens (not shown). Deletions involving the *DNAJB1* and *PRKACA* gene loci were found by aCGH in 11 and 6 of 38 samples, respectively (not shown). In addition, the read-through transcript *GOLT1A-KISS1* was detected by FusionCatcher and at least 1 additional fusion analysis method in 14 of 26 tumors and 2 of 9 normal liver specimens (not shown).

We subsequently analyzed the aCGH data for amplifications and deletions in regions encoding miR sequences. There were 72 miRs altered at the genomic level in at least 25% of primary tumors; all involved deletion of the miR-encoding region, and miR amplifications were far less common (Table I in S1 File). A list of predicted target genes for these commonly deleted miRs was generated using TargetScan[23] and compared against our gene expression data. Target genes with upregulated expression of at least two fold in primary tumors compared with normal liver tissue are shown (Table J in S1 File). Five of the 71 most commonly deleted miRs targeted the *IGF2BP1* gene product, the expression of which was upregulated nearly 100-fold in primary tumors compared with normal liver (Table J in S1 File). *IGF2BP1* is a known pro-tumorigenic factor in traditional hepatocellular carcinoma [26–28]. Additionally, *NTS* is known to be upregulated in FL-HCC [7, 8], and we used TargetScan to generate candidate miRs with at least 7mer binding sites for the *NTS* mRNA transcript. A deletion of at least one of 64 miRs targeting *NTS* was observed in 12 of 17 (71%) primary tumors and 18 of 21 (86%) metastatic tumors (not shown).

## Discussion

We found that genomic amplifications and deletions are common in FL-HCC tumors, and that metastatic tumors have more genomic alterations than primary tumors. It is not unexpected that metastatic tumors had a higher number of amplifications or deletions, as tumor cells in advanced lesions presumably begin with the alterations in the primary tumor and proceed to acquire new genomic changes. However, comparing the specific genomic foci amplified and deleted within individual patients demonstrated significant dissimilarity between matched primary and metastatic tumor samples. Specifically, metastatic tumors retained few of the genomic alterations found in the corresponding primary tumors. These findings suggest that cells with metastatic potential differentiated—and possibly metastasized—from the primary tumor at an early stage, and both primary and metastatic tumor cells then continued to accumulate unique sets of genomic alterations. This is consistent with a “parallel progression model” of metastasis hypothesized in other cancers [29]. These findings could have significant clinical implications as they may imply substantial differences in the response to therapeutics between primary and metastatic FL-HCC tumors.

Nevertheless, our transcriptome data suggest close resemblance among primary and metastatic specimens. As only 3 patients overlap among the 6 with multiple CGH specimens and the 6 with multiple RNA specimens, the discrepancy may simply represent sampling error. More likely, however, is that FL-HCC genomic instability contributes relatively little overall to tumor biology. Primary and metastatic specimens may differ at the chromosomal level, but compensatory mechanisms still result in a similar gene expression profile. This is consistent with our gene-level aCGH and RNA-seq correlation analysis, which demonstrated that the most commonly amplified genes were no more likely to have upregulated RNA expression than commonly deleted genes. From a wider perspective, group similarity is also demonstrated by our finding that fewer than 5% of genes significantly up- or downregulated in primary tumors had fold-changes of opposite magnitude in metastatic specimens.

Prior CGH studies in FL-HCC have been limited by small numbers of patients and the use of lower-resolution, conventional CGH techniques. Marchio *et al*. analyzed 10 FL-HCC tumors, 7 of which demonstrated chromosomal abnormalities [13]. Gains at 1q21–23 were the most frequently seen chromosomal alteration and were found in 6 of 7 cases. Additional reported changes included losses at 8p, 13q, and 14q, as well as gains at 8q and 6p. Furthermore, entire loss of chromosome 18 was found in 3 of the 7 specimens. In our series, amplifications of loci within the same region of 1q were also among the most common alterations, although were present in only 12% and 43% of primary and metastatic tumors, respectively. Meanwhile, entire loss of one copy of chromosome 18 was seen in 2 of 17 primary tumors in our series. Also using conventional CGH arrays, Kakar *et al*. found chromosomal imbalances in 6 of 11 (55%) FL-HCC tumors [14]. Although genomic alterations were evident in all samples in our series, the results of Kakar are similar to our bimodal distribution of chromosomal abnormalities, with 9 of 17 primary tumors (53%) having more than 1,000 gene amplifications or deletions, and the remainder having fewer than 100. It seems likely that the lower-resolution CGH method used by Kaker *et al*. was unable to detect short genomic deletions or amplifications in samples with overall few gene alterations. Although the authors suggest a trend towards improved overall survival in patients without genomic abnormalities, no statistical difference existed in their data, consistent with our findings. Of the 6 samples with abnormalities in the Kakar study, 7p and 7q gains were the most common alteration, seen in 5 and 4 (of 11) patients, respectively. Our data also demonstrate 7p/7q amplifications, albeit at much lower frequencies (fewer than 10% of primary and 30–40% of metastatic tumors).

Contrary to a previous report analyzing *EGFR* copy number by fluorescence in-situ hybridization [30], we did not find *EGFR* gene amplifications in primary tumors, although an amplification was present in 6 of 21 metastatic lesions (not shown). Similarly, our RNA-seq data demonstrated that *EGFR* was not significantly overexpressed in primary or metastatic tumors compared with normal liver. However, members of the EGFR signaling pathway as a whole were significantly upregulated in our pathway analysis. An earlier study also suggested an increase in the RAS, MAPK, and PI3K pathways in 4 tumor samples from 2 FL-HCC patients [31]. In our series, these pathways were not significantly upregulated with the exception of *SHC1*, a member of the RAS-MAPK pathway, which had a mean 4-fold overexpression in primary tumors (not shown). The largest study to date of copy number variations in FL-HCC was described by Cornella *et al*. and included 32 tumor samples.[32] Although we did not see the described broad gains and losses in chromosome 19, our data demonstrated similar rates of amplification at 8q24.3 and deletion at 19p13 and 22q13.

Transcriptional profiling of 14 patients with FL-HCC by Malouf *et al*. demonstrated that of 16 genes significantly upregulated, 4 were the neuroendocrine family genes *PCSK1*, *DNER*, *NTS*, and *CALCA* [16]. Our RNA-seq data confirm upregulation of these genes, from 100- to over 400-fold in primary tumors compared to normal liver. The neuroendocrine characteristics of FL-HCC have been well described, in particular overexpression of neurotensin (NTS) [7, 8]. Intriguingly, we found by aCGH that 30 of 38 tumor specimens had deletion of at least one miR targeting the *NTS* mRNA transcript, correlating with *NTS* overexpression in our gene expression data.

The most consistent biological finding to date in FL-HCC is the recently described *DNAJB1-PRKACA* fusion transcript, which has been detected in all tumor samples (and no normal liver samples) studied thus far [17, 33]. We observed this fusion transcript in 26 of 26 tumor specimens from 15 FL-HCC patients, further strengthening the hypothesis of the *DNAJB1-PRKACA* fusion in tumorigenesis. The significance of the *GOLT1A-KISS1* readthrough transcript we observed in roughly half of tumor specimens remains unclear. Readthrough gene fusions, or transcription-induced chimeras, likely occur in around 4–6% of expressed genes [34, 35], and although recurrent readthrough fusions have been identified in other cancers, their functional role remains to be elucidated [36]. The *GOLT1A-KISS1* chimeric transcript was also detected in 2 of 9 normal liver specimens, decreasing the likelihood of its role in FL-HCC tumor pathogenesis.

Our data also provide insight into miR alterations in FL-HCC, investigations of which have been limited to hypermethylation status of individual miRs [37]. Essentially all miR chromosomal alterations in FL-HCC involve deletion of the miR coding sequence, and as expected, RNA expression of target genes was increased. Tumors had frequent and multiple deletions of miRs with predicted targets for *IGF2BP1*, an mRNA-binding protein with a suspected tumorigenic role in several malignancies, including pancreatic, colorectal, breast, and ovarian cancers [38]. Recently, an anti-apoptotic role for overexpressed IGF2BP1 in conventional HCC was also described [26], as was its putative function in stabilizing the proliferation marker Ki-67 [28]. In our series, the deletion of IGF2BP1-targeting miRs, along with a nearly 100-fold upregulation of IGF2BP1 expression, suggests both a similar role and mechanism for IGF2BP1 upregulation in FL-HCC. Our data also demonstrate a correlating 18-fold upregulation in Ki-67 expression in primary tumors compared with normal liver. Although miR-625 targeting IGF2BP1 has been shown to be frequently downregulated in conventional HCC [27], this miR was deleted in fewer than 5% of our FL-HCC specimens. These results hold promise that potential IGF2BP1-targeted therapeutics could be of clinical benefit in FL-HCC.

In contrast to the one-quarter of conventional HCC tumors that have mutations in the *TP53* gene [39], p53 dysregulation is not evident by *TP53* mutation or methylation status in FL-HCC [40–42]. However, we found evidence of p53 pathway dysregulation by both array CGH and transcriptome data. Of 5 frequently amplified p53 pathway genes in FL-HCC, 4 (*IFR2BP2*, *DUSP1*, *TXNIP*, and *MCL1*) were altered in the expected direction to result in decreased p53 function. Our gene expression data also corroborate p53 pathway inhibition in FL-HCC tumors. The Scian dataset includes 22 genes that are downregulated on p53 activation [43]. Of these 22 genes, 17 were significantly upregulated in our series, suggesting p53 inactivation and decreased p53 activity. Two of the most significantly upregulated of these 17 genes, *CDC20* and *AURKA*, have been identified as therapeutic targets in cancer cell lines having reduced p53 activity [44, 45]. *CDKN3*, *KIF3B*, and *UBE2C*, also among the p53 subset and significantly upregulated in tumors, were previously identified as having putative roles in conventional HCC transformation, proliferation, and invasion, respectively [46–48]. Taken together, the results suggest that despite the lack of *TP53* inactivating mutations in FL-HCC, these tumors are likely able to suppress p53 pathway activity by multiple alternative and potentially targetable means.

In summary, our results elucidate the genomic and transcriptomic landscape of this rare disease. Notable findings include confirmation of the neuroendocrine gene expression profile of FL-HCC and a retained *DNAJB1-PRKACA* fusion transcript. Furthermore, our data indicate a role for p53 pathway inactivation and IGF2BP1 upregulation in FL-HCC, both of which have important roles in conventional HCC pathogenesis. Importantly, the results suggest a mechanism for IGF2BP1 upregulation and implicate potential therapeutic targets involved in p53 pathway inhibition.

## Supporting information

S1 File **Table A.** Most frequently amplified and deleted genetic regions and contained genetic elements of interest. **Table B.** Top upregulated molecular signatures in FL-HCC tumors by aCGH amplifications. **Table C.** Top downregulated molecular signatures in FL-HCC tumors by aCGH deletions.**Table D.** Top 100 significantly dysregulated genes in FL-HCC tumors compared with normal liver. **Table E.** Top upregulated molecular signatures in FL-HCC tumors. **Table F.** Top downregulated molecular signatures in FL-HCC tumors. **Table G.** Top upregulated cancer molecular signatures in FL-HCC tumors. **Table H.** Top downregulated cancer molecular signatures in FL-HCC tumors. **Table I.** Most frequently amplified or deleted miRs in FL-HCC. **Table J.** Upregulated genes with frequent corresponding miR deletions in FL-HCC.
(DOCX)

## Abbreviations

IGF2BP1: (insulin-like growth factor 2 mRNA-binding protein 1)
FL-HCC: (fibrolamellar hepatocellular carcinoma)
aCGH: (array comparative genomic hybridization)
RNA-seq: (RNA sequencing)
miR: (microRNA)
NTS: (neurotensin)
GSA: (gene set analysis)
FFPE: (formalin-fixed, paraffin-embedded)
FF: (fresh-frozen)
PAM: (partitioning around medoids)
