# Supplementary material for: Genome and transcriptome profiling of fibrolamellar hepatocellular carcinoma demonstrates p53 and IGF2BP1 dysregulation
Source: PLoS One. 2017 May 9;12(5):e0176562. doi: 10.1371/journal.pone.0176562 (PMC5423588; doi:10.1371/journal.pone.0176562)
Supplement: S1 File — Table A. Most frequently amplified and deleted genetic regions and contained genetic elements of interest. Table B. Top upregulated molecular signatures in FL-HCC tumors by aCGH amplifications. Table C. Top downregulated molecular signatures in FL-HCC tumors by aCGH deletions.Table D. Top 100 significantly dysregulated genes in FL-HCC tumors compared with normal liver. Table E. Top upregulated molecular signatures in FL-HCC tumors. Table F. Top downregulated molecular signatures in FL-HCC tumors. Table G. Top upregulated cancer molecular signatures in FL-HCC tumors. Table H. Top downregulated cancer molecular signatures in FL-HCC tumors. Table I. Most frequently amplified or deleted miRs in FL-HCC. Table J. Upregulated genes with frequent corresponding miR deletions in FL-HCC. (DOCX) [file pone.0176562.s001.docx]

**Supplementary Data S1 File To:**

**Genome and transcriptome profiling of fibrolamellar hepatocellular carcinoma demonstrates p53 and IGF2BP1 dysregulation**

Eric C. Sorenson^1^, Raya Khanin^2^, Zubin M. Bamboat^1^, Michael J. Cavnar^1^, Teresa S. Kim^1^, Eran Sadot^1^, Shan Zeng^1^, Jonathan B. Greer^1^, Adrian M. Seifert^1^, Noah A. Cohen^1^, Megan H. Crawley^1^, Benjamin L. Green^1^, David S. Klimstra,^3^ Ronald P. DeMatteo^1^

From the Departments of Surgery,^1^ Computational Biology,^2^ and Pathology,^3^ Memorial Sloan Kettering Cancer Center, New York City, U.S.A.

**Contents Page**

Table A in S1 File 2

Table B in S1 File 3

Table C in S1 File 3

Table D in S1 File 4

Table E in S1 File 6

Table F in S1 File 7

Table G in S1 File 8

Table H in S1 File 8

Table I in S1 File 9

Table J in S1 File 11

**Table A in S1 File.** **Most frequently amplified and deleted genetic regions and contained genetic elements of interest.**

|  | | |  | |  |  |  | Fraction of samples with alteration | | | |
| --- | --- | --- | --- | --- | --- | --- | --- | --- | --- | --- | --- |
|  | | | |  |  |  |  | Amplification | | Deletion | |
| Locus | | | | Region | Size (kb) | Number of genes | Genetic elements of interest | Primary | Metastatic | Primary | Metastatic |
| **Gain** |  | | | | | | | | | | |
| 7p14.1 | | 38270942-38332055 | | | 61 | 1 | TARP | 0.47 | 0.81 | 0.00 | 0.00 |
| 16q22.1 | | 70154957-70185917 | | | 31 | 1 | TAT | 0.29 | 0.19 | 0.00 | 0.00 |
| 11q24.2 | | 73082262-73091980 | | | 10 | 3 | OR4P4, OR4S2, OR4C6 | 0.24 | 0.57 | 0.18 | 0.00 |
| 11p12.1 | | 54037199-54042734 | | | 6 | 1 | OR4C11 | 0.24 | 0.52 | 0.18 | 0.00 |
| 6q24.2 | | 88067179-88106572 | | | 39 | 1 | C6orf142 | 0.24 | 0.33 | 0.12 | 0.05 |
| 16q11 | | 55161177-55207305 | | | 46 | 1 | VAC14 | 0.24 | 0.24 | 0.00 | 0.00 |
| 14q11 | | 55127355-55131703 | | | 4 | 1 | HEATR4 | 0.24 | 0.19 | 0.00 | 0.10 |
| 16q22.1 | | 69333748-69343392 | | | 10 | 2 | ANKRD11, SPG7 | 0.24 | 0.14 | 0.00 | 0.05 |
| 16q22.1 | | 70220613-70226181 | | | 6 | 1 | MARVELD3 | 0.24 | 0.14 | 0.00 | 0.00 |
| 8p11.22 | | 39354149-39499752 | | | 146 | 2 | ADAM5P, ADAM3A | 0.18 | 0.57 | 0.12 | 0.19 |
| 5q21.3 | | 150839754-150844444 | | | 5 | 1 | SLC12A7 | 0.18 | 0.52 | 0.00 | 0.00 |
| 1p15.33 | | 1155105-1166192 | | | 11 | 1 | LCE3C | 0.18 | 0.43 | 0.18 | 0.10 |
| 1q42.2 | | 232791749-233314326 | | | 523 | 1 | IRF2BP2 | 0.18 | 0.43 | 0.00 | 0.00 |
| 5q35.1 | | 172126507-172134087 | | | 8 | 1 | DUSP1 | 0.18 | 0.43 | 0.00 | 0.00 |
| 8q24.3 | | 145081795-145112543 | | | 31 | 2 | hsa-mir-661, PLEC1 | 0.12 | 0.48 | 0.06 | 0.00 |
| 1q24.3 | | 145113549-145154888 | | | 41 | 3 | TXNIP, POLR3GL, ANKRD34A | 0.12 | 0.43 | 0.00 | 0.00 |
| 1q21.1 | | 144131305-144183050 | | | 52 | 1 | MCL1 | 0.12 | 0.43 | 0.00 | 0.00 |
| 8q21.2 | | 148804083-148819378 | | | 15 | 2 | PARP10, GRINA | 0.12 | 0.43 | 0.06 | 0.00 |
| 8q11.21 | | 48573822-48603374 | | | 30 | 1 | KIAA0146 | 0.06 | 0.48 | 0.00 | 0.00 |
| 8q22.3 | | 105517672-105538262 | | | 21 | 1 | DPYS | 0.06 | 0.43 | 0.00 | 0.00 |
|  | |  | | |  |  |  |  |  |  |  |
| **Loss** | |  | | |  |  |  |  |  |  |  |
| 10q11.22 | | 46384985-46558272 | | | 173 | 4 | SYT15, GPRIN2, PPYR1 | 0.00 | 0.05 | 0.53 | 0.29 |
| 10q26.2 | | 127566573-127599309 | | | 33 | 1 | FANK1 | 0.00 | 0.00 | 0.53 | 0.14 |
| 15p11.1 | | 18831600-19430207 | | | 599 | 2 | HERC2P3, POTEB | 0.12 | 0.14 | 0.53 | 0.48 |
| 21p11.2 | | 9882922-10136303 | | | 253 | 6 | BAGE, TPTE | 0.00 | 0.05 | 0.53 | 0.43 |
| 3p12.3 | | 75783255-75803353 | | | 20 | 1 | FRG2C | 0.00 | 0.00 | 0.53 | 0.29 |
| 4q35.2 | | 191111451-191153613 | | | 42 | 2 | FRG1, TUBB4Q | 0.00 | 0.00 | 0.53 | 0.48 |
| 15q11.1 | | 19845942-19983398 | | | 138 | 2 | OR4M2 | 0.06 | 0.14 | 0.47 | 0.48 |
| 4p16.3 | | 34020-65688 | | | 32 | 2 | ZNF595, ZNF718 | 0.06 | 0.05 | 0.47 | 0.24 |
| 6p11.2 | | 57466683-57683389 | | | 217 | 1 | PRIM2 | 0.00 | 0.00 | 0.47 | 0.14 |
| 6p25.3 | | 241674-297953 | | | 56 | 1 | DUSP22 | 0.06 | 0.14 | 0.47 | 0.38 |
| 22q11.1 | | 16654348-17274339 | | | 620 | 7 | PEX26, USP18 | 0.00 | 0.00 | 0.29 | 0.52 |
| 22q11.21 | | 17274835-22735036 | | | 5460 | 90 | PRODH, SLC25A1, MAPK1, BCR, MIF | 0.00 | 0.00 | 0.29 | 0.52 |
| 22q11.1 | | 15448186-17274339 | | | 1826 | 13 | IL17RA, BID | 0.00 | 0.00 | 0.24 | 0.52 |
| 22q13.1-13.2 | | 39922709-44213320 | | | 4291 | 73 | hsa-mir-33a, ST13. EP300 | 0.00 | 0.00 | 0.24 | 0.52 |
| 22q13.31-13.32 | | 44216958-49571068 | | | 5354 | 48 | GRAMD4, WNT7B, IL17REL | 0.00 | 0.00 | 0.24 | 0.57 |

**Table B in S1 File. Top upregulated molecular signatures in FL-HCC tumors by aCGH amplifications.**

| Pathway Name | Genes in pathway | Pathway genes amplified in dataset | Adjusted p-value |
| --- | --- | --- | --- |
| CASTELLANO_NRAS_TARGETS_DN | 14 | 11 | <0.0001 |
| CROONQUIST_NRAS_VS_STROMAL_STIMULATION_DN | 99 | 77 | <0.0001 |
| CROONQUIST_NRAS_SIGNALING_DN | 72 | 58 | <0.0001 |
| JI_CARCINOGENESIS_BY_KRAS_AND_STK11_DN | 17 | 11 | <0.0001 |
| SCHAEFFER_PROSTATE_DEVELOPMENT_AND_CANCER_BOX6_UP | 8 | 8 | <0.0001 |
| MIKKELSEN_DEDIFFERENTIATED_STATE_DN | 7 | 4 | <0.0001 |
| VERRECCHIA_RESPONSE_TO_TGFB1_C2 | 25 | 17 | <0.0001 |
| VERRECCHIA_RESPONSE_TO_TGFB1_C4 | 13 | 8 | <0.0001 |
| VERRECCHIA_RESPONSE_TO_TGFB1_C1 | 19 | 12 | <0.0001 |
| VERRECCHIA_EARLY_RESPONSE_TO_TGFB1 | 58 | 39 | <0.0001 |
| REACTOME_UNWINDING_OF_DNA | 11 | 10 | <0.0001 |
| REGULATION_OF_DNA_REPLICATION | 20 | 15 | <0.0001 |
| CHROMOSOME_CONDENSATION | 10 | 7 | <0.0001 |
| REACTOME_SIGNALING_BY_NOTCH3 | 12 | 11 | <0.0001 |
| REACTOME_RECEPTOR_LIGAND_BINDING_INITIATES_THE_SECOND | 11 | 9 | <0.0001 |
| _PROTEOLYTIC_CLEAVAGE_OF_NOTCH_RECEPTOR |  |  | <0.0001 |
| REACTOME_SIGNALING_BY_NOTCH4 | 12 | 11 | <0.0001 |
| REACTOME_SIGNALING_BY_NOTCH2 | 12 | 10 | <0.0001 |
| XU_HGF_SIGNALING_NOT_VIA_AKT1_48HR_DN | 20 | 15 | <0.0001 |
| REACTOME_SIGNALING_BY_NOTCH3 | 12 | 11 | <0.0001 |
| REACTOME_SHC1_EVENTS_IN_EGFR_SIGNALING | 15 | 13 | <0.0001 |
| REACTOME_SIGNALING_BY_NOTCH4 | 12 | 11 | <0.0001 |
| CROONQUIST_NRAS_SIGNALING_DN | 72 | 58 | <0.0001 |
| REACTOME_SIGNALING_BY_NOTCH2 | 12  59 | 10 | <0.0001 |
| DASU_IL6_SIGNALING_UP | 59 | 41 | <0.0001 |
| REACTOME_SIGNALING_BY_CONSTITUTIVELY_ACTIVE_EGFR | 17 | 14 | <0.0001 |
| CREIGHTON_AKT1_SIGNALING_VIA_MTOR_DN | 23 | 17 | <0.0001 |
| REACTOME_SHC_MEDIATED_SIGNALLING | 15 | 12 | <0.0001 |
| SCHAVOLT_TARGETS_OF_TP53_AND_TP63 | 16 | 11 | <0.0001 |
| NUCLEOBASENUCLEOSIDENUCLEOTIDE_KINASE_ACTIVITY | 25 | 17 | <0.0001 |

**Table C in S1 File. Top downregulated molecular signatures in FL-HCC tumors by aCGH deletions.**

| Pathway Name | Genes in pathway | Pathway genes deleted in dataset | Adjusted p-value |
| --- | --- | --- | --- |
| REACTOME_CYTOCHROME_P450_ARRANGED_BY_SUBSTRATE_TYPE | 50 | 40 | <0.0001 |
| KEGG_DRUG_METABOLISM_CYTOCHROME_P450 | 72 | 56 | <0.0001 |
| KEGG_METABOLISM_OF_XENOBIOTICS_BY_CYTOCHROME_P450 | 70 | 51 | <0.0001 |
| REGULATION_OF_IMMUNE_EFFECTOR_PROCESS | 15 | 11 | <0.0001 |

**Table D in S1 File.** **Top 100 significantly dysregulated genes in FL-HCC tumors compared with normal liver.**

| Gene Symbol | adj. p-value | Fold Change | Mean Expression Normal Liver | Mean Expression Tumor |
| --- | --- | --- | --- | --- |
| MUC13 | 1.3E-98 | 539 | 91 | 49269 |
| PCSK1 | 5.4E-96 | 489 | 42 | 20674 |
| PAEP | 2.3E-95 | 736 | 80 | 58973 |
| CPLX2 | 5.9E-93 | 661 | 24 | 15567 |
| TMEM163 | 1.4E-75 | 187 | 27 | 4979 |
| CA12 | 2.6E-67 | 393 | 43 | 17038 |
| PPP2R2C | 6.3E-66 | 435 | 1 | 382 |
| SLC16A14 | 2.8E-65 | 118 | 115 | 13557 |
| C10orf90 | 1.4E-62 | 125 | 6 | 768 |
| TPPP2 | 9.5E-56 | -64 | 490 | 8 |
| ADRA1A | 1.1E-55 | -54 | 2766 | 52 |
| USH1C | 5.1E-52 | 51 | 93 | 4751 |
| DNER | 2.0E-51 | 416 | 9 | 3844 |
| TGM3 | 5.4E-51 | 188 | 11 | 1979 |
| SERHL2 | 5.7E-48 | 43 | 68 | 2890 |
| NRG2 | 2.0E-47 | 45 | 18 | 804 |
| PAK3 | 2.9E-47 | 282 | 9 | 2430 |
| SCG2 | 4.3E-45 | 152 | 2 | 348 |
| TESC | 1.3E-44 | 92 | 177 | 16326 |
| NUGGC | 3.2E-44 | -30 | 1848 | 61 |
| ZNF385D | 7.5E-44 | 59 | 10 | 566 |
| GNAZ | 2.7E-43 | 34 | 30 | 1024 |
| CTNNA3 | 9.9E-43 | -49 | 212 | 4 |
| SLCO4C1 | 6.8E-41 | -54 | 354 | 7 |
| GPR128 | 8.1E-41 | -33 | 1137 | 34 |
| FXYD2 | 8.2E-41 | 28 | 282 | 7873 |
| ADCY1 | 1.3E-40 | -33 | 7198 | 221 |
| TRIM31 | 4.4E-40 | 152 | 12 | 1824 |
| SHBG | 2.0E-38 | -21 | 1839 | 86 |
| NEB | 2.1E-37 | 24 | 208 | 4891 |
| ASPHD1 | 6.5E-37 | 77 | 53 | 4068 |
| OIT3 | 1.7E-36 | -15 | 5299 | 353 |
| FADS6 | 5.0E-36 | -27 | 362 | 13 |
| SLC3A1 | 1.9E-35 | -37 | 1250 | 34 |
| SLCO1C1 | 3.0E-34 | 51 | 4 | 224 |
| TTC39A | 9.6E-34 | 20 | 39 | 782 |
| RNF165 | 1.0E-33 | -14 | 911 | 65 |
| ZNF703 | 1.1E-33 | 19 | 327 | 6075 |
| EXPH5 | 1.3E-33 | -17 | 1221 | 73 |
| DPP4 | 1.6E-33 | -15 | 2486 | 166 |
| PGLYRP2 | 2.5E-33 | -33 | 16572 | 507 |
| GATM | 1.4E-32 | -31 | 89496 | 2920 |
| CATSPERB | 1.4E-32 | 36 | 22 | 799 |
| NOX4 | 3.6E-32 | 21 | 18 | 365 |
| VIPR1 | 3.9E-32 | -15 | 3597 | 248 |
| LPA | 5.4E-32 | -52 | 10425 | 200 |
| VASH2 | 6.8E-32 | 23 | 31 | 726 |
| FAM155B | 2.0E-31 | 22 | 10 | 220 |
| GUCY2C | 3.5E-31 | 89 | 11 | 1027 |
| ACSM5 | 5.6E-31 | -17 | 14895 | 898 |
| HLF | 7.6E-31 | -12 | 9636 | 800 |
| TTYH2 | 1.0E-30 | 16 | 179 | 2783 |
| SALL4 | 1.0E-30 | -13 | 205 | 15 |
| HSD3B2 | 1.3E-30 | -28 | 62 | 2 |
| DIO1 | 2.6E-30 | -25 | 6099 | 247 |
| NETO2 | 2.7E-30 | 16 | 50 | 817 |
| GOLGA6B | 3.7E-30 | -56 | 82 | 1 |
| GYS2 | 4.3E-30 | -15 | 9940 | 644 |
| SLC22A9 | 5.5E-30 | -12 | 1517 | 123 |
| IGF2BP3 | 5.6E-30 | 23 | 6 | 137 |
| AUTS2 | 8.4E-30 | -11 | 4211 | 392 |
| CUX2 | 9.8E-30 | -28 | 5131 | 184 |
| KCND3 | 1.1E-29 | -13 | 1693 | 130 |
| ACSL1 | 1.2E-29 | -12 | 86821 | 7514 |
| APOM | 2.1E-29 | -11 | 16182 | 1505 |
| GULP1 | 3.6E-29 | 33 | 41 | 1350 |
| SOX5 | 4.6E-29 | -13 | 881 | 68 |
| AADAT | 6.1E-29 | -11 | 2402 | 214 |
| GBP7 | 1.8E-28 | -15 | 3012 | 199 |
| DPP10 | 1.9E-28 | 16 | 19 | 297 |
| ACSM2B | 2.4E-28 | -11 | 30503 | 2841 |
| XDH | 3.8E-28 | -14 | 6640 | 483 |
| ADAMTS13 | 5.0E-28 | -10 | 5566 | 576 |
| ART4 | 1.2E-27 | -16 | 1676 | 108 |
| MLIP | 1.5E-27 | -18 | 1145 | 64 |
| RGPD3 | 1.5E-27 | -11 | 326 | 31 |
| TMPRSS6 | 1.5E-27 | -14 | 10210 | 725 |
| GOLM1 | 1.6E-27 | 12 | 752 | 9293 |
| ACSM2A | 2.7E-27 | -13 | 35515 | 2802 |
| IQGAP3 | 1.6E-26 | 21 | 49 | 1015 |
| CPEB3 | 1.7E-26 | -10 | 2427 | 249 |
| SYDE2 | 2.1E-26 | -18 | 317 | 18 |
| ABCA6 | 2.6E-26 | -10 | 6930 | 704 |
| KLKB1 | 2.8E-26 | -9 | 13770 | 1451 |
| SLC2A2 | 3.4E-26 | -15 | 26002 | 1741 |
| MYO15A | 4.4E-26 | -10 | 472 | 46 |
| GOLGA6A | 4.6E-26 | -33 | 402 | 12 |
| PDE1C | 4.8E-26 | 104 | 23 | 2423 |
| ITIH1 | 4.9E-26 | -12 | 140090 | 11659 |
| AFM | 5.5E-26 | -13 | 32339 | 2410 |
| PDE10A | 1.0E-25 | 12 | 92 | 1070 |
| PBK | 1.1E-25 | 43 | 10 | 408 |
| NIPAL1 | 1.1E-25 | -13 | 775 | 59 |
| OXT | 1.3E-25 | -51 | 145 | 3 |
| CYBA | 1.5E-25 | 11 | 2694 | 30385 |
| GPR125 | 1.6E-25 | -9 | 8415 | 961 |
| BCAN | 1.8E-25 | 35 | 3 | 113 |
| CPE | 1.8E-25 | 20 | 586 | 11577 |
| SARDH | 1.9E-25 | -9 | 13029 | 1399 |
| HHIPL2 | 2.6E-25 | 18 | 9 | 168 |

**Table E in S1 File. Top upregulated molecular signatures in FL-HCC tumors.**

| Pathway Name | Genes in pathway | Pathway genes upregulated in dataset | Adjusted p-value |
| --- | --- | --- | --- |
| FARMER_BREAST_CANCER_CLUSTER_5 | 19 | 17 | <0.0001 |
| ANASTASSIOU_CANCER_MESENCHYMAL_TRANSITION_SIGNATURE | 63 | 53 | <0.0001 |
| 3_5_CYCLIC_NUCLEOTIDE_PHOSPHODIESTERASE_ACTIVITY | 13 | 12 | <0.0001 |
| COLLAGEN | 23 | 17 | <0.0001 |
| LU_TUMOR_ENDOTHELIAL_MARKERS_UP | 22 | 16 | <0.0001 |
| LU_TUMOR_VASCULATURE_UP | 29 | 22 | <0.0001 |
| REACTOME_RNA_POL_I_PROMOTER_OPENING | 26 | 23 | <0.0001 |
| SU_PLACENTA | 18 | 14 | <0.0001 |
| REACTOME_PACKAGING_OF_TELOMERE_ENDS | 24 | 23 | <0.0001 |
| MONTERO_THYROID_CANCER_POOR_SURVIVAL_UP | 12 | 12 | <0.0001 |
| MAHADEVAN_GIST_MORPHOLOGICAL_SWITCH | 15 | 11 | <0.0001 |
| FINETTI_BREAST_CANCER_KINOME_RED | 16 | 15 | <0.0001 |
| EXTRACELLULAR_MATRIX_STRUCTURAL_CONSTITUENT | 21 | 19 | <0.0001 |
| YAGUE_PRETUMOR_DRUG_RESISTANCE_DN | 13 | 9 | <0.0001 |
| TURASHVILI_BREAST_DUCTAL_CARCINOMA_VS_DUCTAL_NORMAL_UP | 42 | 32 | <0.0001 |
| REACTOME_DEPOSITION_OF_NEW_CENPA_CONTAINING_NUCLEOSOMES_AT_THE_CENTROMERE | 37 | 34 | <0.0001 |
| NIELSEN_GIST_VS_SYNOVIAL_SARCOMA_UP | 14 | 9 | <0.0001 |
| THUM_MIR21_TARGETS_HEART_DISEASE_UP | 16 | 14 | <0.0001 |
| NAKAYAMA_SOFT_TISSUE_TUMORS_PCA2_UP | 75 | 63 | <0.0001 |
| REACTOME_KERATAN_SULFATE_DEGRADATION | 10 | 10 | <0.0001 |
| TURASHVILI_BREAST_LOBULAR_CARCINOMA_VS_LOBULAR_NORMAL_DN | 74 | 48 | <0.0001 |
| SMID_BREAST_CANCER_LUMINAL_A_DN | 16 | 13 | <0.0001 |
| CLAUS_PGR_POSITIVE_MENINGIOMA_DN | 12 | 10 | <0.0001 |
| BIOCARTA_VITCB_PATHWAY | 11 | 6 | <0.0001 |
| LU_TUMOR_ANGIOGENESIS_UP | 25 | 20 | <0.0001 |
| TURASHVILI_BREAST_LOBULAR_CARCINOMA_VS_DUCTAL_NORMAL_UP | 67 | 48 | <0.0001 |
| PID_SYNDECAN_1_PATHWAY | 45 | 32 | <0.0001 |
| LIEN_BREAST_CARCINOMA_METAPLASTIC | 34 | 26 | <0.0001 |
| ROY_WOUND_BLOOD_VESSEL_UP | 49 | 37 | <0.0001 |
| REACTOME_MEIOTIC_RECOMBINATION | 43 | 33 | <0.0001 |
| KUMAMOTO_RESPONSE_TO_NUTLIN_3A_DN | 10 | 8 | <0.0001 |
| LIAO_HAVE_SOX4_BINDING_SITES | 40 | 29 | <0.0001 |
| NEWMAN_ERCC6_TARGETS_DN | 33 | 24 | <0.0001 |
| ZHAN_MULTIPLE_MYELOMA_PR_UP | 41 | 37 | <0.0001 |
| XU_HGF_SIGNALING_NOT_VIA_AKT1_48HR_DN | 20 | 15 | <0.0001 |
| GREENBAUM_E2A_TARGETS_UP | 33 | 30 | <0.0001 |
| LY_AGING_MIDDLE_DN | 16 | 14 | <0.0001 |
| TRAYNOR_RETT_SYNDROM_DN | 16 | 11 | <0.0001 |
| KANG_DOXORUBICIN_RESISTANCE_UP | 53 | 44 | <0.0001 |
| KRCTCNNNNMANAGC_UNKNOWN | 29 | 21 | <0.0001 |

**Table F in S1 File. Top downregulated molecular signatures in FL-HCC tumors.**

| Pathway Name | Genes in pathway | Pathway genes downregulated in dataset | adjusted p-value |
| --- | --- | --- | --- |
| REACTOME_XENOBIOTICS | 14 | 14 | <0.0001 |
| LEE_LIVER_CANCER | 47 | 46 | <0.0001 |
| REACTOME_SYNTHESIS_OF_BILE_ACIDS_AND_BILE_SALTS_VIA_24_HYDROXYCHOLESTEROL | 10 | 10 | <0.0001 |
| REACTOME_RECYCLING_OF_BILE_ACIDS_AND_SALTS | 10 | 9 | <0.0001 |
| OXYGEN_BINDING | 20 | 15 | <0.0001 |
| REACTOME_SYNTHESIS_OF_BILE_ACIDS_AND_BILE_SALTS_VIA_7ALPHA_HYDROXYCHOLESTEROL | 15 | 14 | <0.0001 |
| KEGG_RETINOL_METABOLISM | 55 | 46 | <0.0001 |
| REACTOME_CYTOCHROME_P450_ARRANGED_BY_SUBSTRATE_TYPE | 43 | 36 | <0.0001 |
| SU_LIVER | 54 | 48 | <0.0001 |
| REACTOME_BILE_ACID_AND_BILE_SALT_METABOLISM | 26 | 23 | <0.0001 |
| KEGG_PRIMARY_BILE_ACID_BIOSYNTHESIS | 16 | 15 | <0.0001 |
| REACTOME_SYNTHESIS_OF_BILE_ACIDS_AND_BILE_SALTS | 19 | 17 | <0.0001 |
| CHIANG_LIVER_CANCER_SUBCLASS_PROLIFERATION_DN | 174 | 164 | <0.0001 |
| CAIRO_HEPATOBLASTOMA_DN | 262 | 242 | <0.0001 |
| KEGG_DRUG_METABOLISM_CYTOCHROME_P450 | 64 | 51 | <0.0001 |
| BIOCARTA_NUCLEARRS_PATHWAY | 14 | 12 | <0.0001 |
| REACTOME_PHASE1_FUNCTIONALIZATION_OF_COMPOUNDS | 61 | 49 | <0.0001 |
| WENG_POR_TARGETS_GLOBAL_UP | 18 | 16 | <0.0001 |
| CHIANG_LIVER_CANCER_SUBCLASS_POLYSOMY7_UP | 74 | 66 | <0.0001 |
| YAMASHITA_LIVER_CANCER_STEM_CELL_DN | 76 | 62 | <0.0001 |
| YAMASHITA_LIVER_CANCER_WITH_EPCAM_DN | 15 | 13 | <0.0001 |
| MONOCARBOXYLIC_ACID_TRANSMEMBRANE_TRANSPORTER_ACTIVITY | 10 | 8 | <0.0001 |
| SUMI_HNF4A_TARGETS | 29 | 26 | <0.0001 |
| KEGG_GLYCINE_SERINE_AND_THREONINE_METABOLISM | 30 | 26 | <0.0001 |
| KEGG_METABOLISM_OF_XENOBIOTICS_BY_CYTOCHROME_P450 | 60 | 46 | <0.0001 |
| BIOCARTA_LECTIN_PATHWAY | 12 | 12 | <0.0001 |
| DETECTION_OF_CHEMICAL_STIMULUS | 13 | 11 | <0.0001 |
| HSIAO_LIVER_SPECIFIC_GENES | 243 | 211 | <0.0001 |
| BOYAULT_LIVER_CANCER_SUBCLASS_G123_DN | 50 | 45 | <0.0001 |
| REACTOME_AMINE_LIGAND_BINDING_RECEPTORS | 13 | 8 | <0.0001 |
| TUOMISTO_TUMOR_SUPPRESSION_BY_COL13A1_DN | 14 | 10 | <0.0001 |
| ICHIBA_GRAFT_VERSUS_HOST_DISEASE_35D_DN | 45 | 33 | <0.0001 |
| CHIANG_LIVER_CANCER_SUBCLASS_UNANNOTATED_UP | 80 | 73 | <0.0001 |
| KEGG_DRUG_METABOLISM_OTHER_ENZYMES | 47 | 38 | <0.0001 |
| chr3q24 | 14 | 12 | <0.0001 |
| OHGUCHI_LIVER_HNF4A_TARGETS_DN | 137 | 118 | <0.0001 |
| BIOCARTA_CLASSIC_PATHWAY | 14 | 14 | <0.0001 |
| BIOCARTA_COMP_PATHWAY | 19 | 19 | <0.0001 |
| BOYAULT_LIVER_CANCER_SUBCLASS_G12_DN | 14 | 12 | <0.0001 |
| LEE_LIVER_CANCER_SURVIVAL_UP | 171 | 151 | <0.0001 |

**Table G in S1 File. Top upregulated cancer molecular signatures in FL-HCC tumors.**

| Pathway Name | Genes in pathway | Pathway genes upregulated in dataset | adjusted p-value |
| --- | --- | --- | --- |
| CROONQUIST_NRAS_VS_STROMAL_STIMULATION_DN | 95 | 72 | <0.0001 |
| CROONQUIST_NRAS_SIGNALING_DN | 71 | 54 | <0.0001 |
| EGUCHI_CELL_CYCLE_RB1_TARGETS | 22 | 18 | <0.0001 |
| SCIAN_CELL_CYCLE_TARGETS_OF_TP53_AND_TP73_DN | 22 | 17 | <0.0001 |
| REACTOME_SIGNALING_BY_NOTCH3 | 12 | 10 | <0.0001 |
| REACTOME_RECEPTOR_LIGAND_BINDING_INITIATES_THE_SECOND_PROTEOLYTIC_CLEAVAGE_OF_NOTCH_RECEPTOR | 11 | 9 | <0.0001 |
| XU_HGF_SIGNALING_NOT_VIA_AKT1_48HR_DN | 20 | 15 | <0.0001 |
| REACTOME_SHC1_EVENTS_IN_EGFR_SIGNALING | 15 | 9 | <0.0001 |
| REACTOME_SHC1_EVENTS_IN_ERBB4_SIGNALING | 19 | 11 | <0.0001 |
| SCHAVOLT_TARGETS_OF_TP53_AND_TP63 | 15 | 11 | <0.0001 |
| GENTLES_LEUKEMIC_STEM_CELL_DN | 10 | 6 | 0.05 |
| REACTOME_SIGNALING_BY_NOTCH2 | 12 | 9 | 0.05 |
| REACTOME_GRB2_EVENTS_IN_ERBB2_SIGNALING | 21 | 12 | 0.05 |
| REACTOME_SIGNALING_BY_NOTCH4 | 12 | 10 | 0.05 |

**Table H in S1 File. Top downregulated cancer molecular signatures in FL-HCC tumors.**

| Pathway Name | Genes in pathway | Pathway genes downregulated in dataset | adjusted p-value |
| --- | --- | --- | --- |
| CROONQUIST_NRAS_SIGNALING_UP | 39 | 29 | <0.0001 |
| LEE_LIVER_CANCER_MYC_TGFA_DN | 56 | 42 | <0.0001 |
| COULOUARN_TEMPORAL_TGFB1_SIGNATURE_DN | 129 | 94 | <0.0001 |
| KEGG_PPAR_SIGNALING_PATHWAY | 62 | 47 | <0.0001 |
| ZHENG_IL22_SIGNALING_UP | 46 | 33 | <0.0001 |
| KEGG_TYROSINE_METABOLISM | 36 | 27 | <0.0001 |

**Table I in S1 File. Most frequently amplified or deleted miRs in FL-HCC.**

|  |  | primary tumors | | recurrences / metastases | |
| --- | --- | --- | --- | --- | --- |
| miR | Chromosome location | frequency deleted | frequency amplified | frequency deleted | frequency amplified |
| hsa-mir-570 | 3q29 | 0.35 | 0.06 | 0.43 | 0.00 |
| hsa-mir-1227 | 19p13.3 | 0.29 | 0.00 | 0.00 | 0.05 |
| hsa-mir-637 | 19p13.3 | 0.29 | 0.00 | 0.19 | 0.05 |
| hsa-mir-7-3 | 19p13.3 | 0.29 | 0.00 | 0.19 | 0.05 |
| hsa-mir-220b | 19p13.3 | 0.29 | 0.00 | 0.14 | 0.05 |
| hsa-mir-1238 | 19p13.2 | 0.29 | 0.00 | 0.10 | 0.05 |
| hsa-mir-638 | 19p13.2 | 0.29 | 0.00 | 0.10 | 0.05 |
| hsa-mir-199a-1 | 19p13.2 | 0.29 | 0.00 | 0.10 | 0.05 |
| hsa-mir-24-2 | 19p13.12 | 0.29 | 0.00 | 0.05 | 0.24 |
| hsa-mir-27a | 19p13.12 | 0.29 | 0.00 | 0.05 | 0.24 |
| hsa-mir-23a | 19p13.12 | 0.29 | 0.00 | 0.05 | 0.24 |
| hsa-mir-181c | 19p13.12 | 0.29 | 0.00 | 0.05 | 0.05 |
| hsa-mir-181d | 19p13.12 | 0.29 | 0.00 | 0.05 | 0.05 |
| hsa-mir-99b | 19q13.41 | 0.29 | 0.00 | 0.19 | 0.00 |
| hsa-let-7e | 19q13.41 | 0.29 | 0.00 | 0.19 | 0.00 |
| hsa-mir-125a | 19q13.41 | 0.29 | 0.00 | 0.19 | 0.00 |
| hsa-mir-643 | 19q13.41 | 0.29 | 0.00 | 0.19 | 0.00 |
| hsa-mir-512-1 | 19q13.42 | 0.29 | 0.00 | 0.19 | 0.00 |
| hsa-mir-512-2 | 19q13.42 | 0.29 | 0.00 | 0.19 | 0.00 |
| hsa-mir-498 | 19q13.42 | 0.29 | 0.00 | 0.19 | 0.00 |
| hsa-mir-520e | 19q13.42 | 0.29 | 0.00 | 0.19 | 0.00 |
| hsa-mir-515-1 | 19q13.42 | 0.29 | 0.00 | 0.19 | 0.00 |
| hsa-mir-519e | 19q13.42 | 0.29 | 0.00 | 0.19 | 0.00 |
| hsa-mir-520f | 19q13.42 | 0.29 | 0.00 | 0.19 | 0.00 |
| hsa-mir-515-2 | 19q13.42 | 0.29 | 0.00 | 0.19 | 0.00 |
| hsa-mir-519c | 19q13.42 | 0.29 | 0.00 | 0.19 | 0.00 |
| hsa-mir-520a | 19q13.42 | 0.29 | 0.00 | 0.19 | 0.00 |
| hsa-mir-526b | 19q13.42 | 0.29 | 0.00 | 0.19 | 0.00 |
| hsa-mir-519b | 19q13.42 | 0.29 | 0.00 | 0.19 | 0.00 |
| hsa-mir-525 | 19q13.42 | 0.29 | 0.00 | 0.19 | 0.00 |
| hsa-mir-523 | 19q13.42 | 0.29 | 0.00 | 0.19 | 0.00 |
| hsa-mir-518f | 19q13.42 | 0.29 | 0.00 | 0.19 | 0.00 |
| hsa-mir-520b | 19q13.42 | 0.29 | 0.00 | 0.19 | 0.00 |
| hsa-mir-518b | 19q13.42 | 0.29 | 0.00 | 0.19 | 0.00 |
| hsa-mir-526a-1 | 19q13.42 | 0.29 | 0.00 | 0.19 | 0.00 |
| hsa-mir-520c | 19q13.42 | 0.29 | 0.00 | 0.19 | 0.00 |
| hsa-mir-518c | 19q13.42 | 0.29 | 0.00 | 0.19 | 0.00 |
| hsa-mir-524 | 19q13.42 | 0.29 | 0.00 | 0.19 | 0.00 |
| hsa-mir-517a | 19q13.42 | 0.29 | 0.00 | 0.19 | 0.00 |
| hsa-mir-519d | 19q13.42 | 0.29 | 0.00 | 0.19 | 0.00 |
| hsa-mir-521-2 | 19q13.42 | 0.29 | 0.00 | 0.19 | 0.00 |
| hsa-mir-520d | 19q13.42 | 0.29 | 0.00 | 0.19 | 0.00 |
| hsa-mir-517b | 19q13.42 | 0.29 | 0.00 | 0.19 | 0.00 |
| hsa-mir-520g | 19q13.42 | 0.29 | 0.00 | 0.19 | 0.00 |
| hsa-mir-516b-2 | 19q13.42 | 0.29 | 0.00 | 0.19 | 0.00 |
| hsa-mir-526a-2 | 19q13.42 | 0.29 | 0.00 | 0.19 | 0.00 |
| hsa-mir-518e | 19q13.42 | 0.29 | 0.00 | 0.19 | 0.00 |
| hsa-mir-518a-1 | 19q13.42 | 0.29 | 0.00 | 0.19 | 0.00 |
| hsa-mir-518d | 19q13.42 | 0.29 | 0.00 | 0.19 | 0.00 |
| hsa-mir-516b-1 | 19q13.42 | 0.29 | 0.00 | 0.19 | 0.00 |
| hsa-mir-518a-2 | 19q13.42 | 0.29 | 0.00 | 0.19 | 0.00 |
| hsa-mir-517c | 19q13.42 | 0.29 | 0.00 | 0.19 | 0.00 |
| hsa-mir-520h | 19q13.42 | 0.29 | 0.00 | 0.19 | 0.00 |
| hsa-mir-521-1 | 19q13.42 | 0.29 | 0.00 | 0.19 | 0.05 |
| hsa-mir-522 | 19q13.42 | 0.29 | 0.00 | 0.19 | 0.05 |
| hsa-mir-519a-1 | 19q13.42 | 0.29 | 0.00 | 0.19 | 0.05 |
| hsa-mir-527 | 19q13.42 | 0.29 | 0.00 | 0.19 | 0.00 |
| hsa-mir-516a-1 | 19q13.42 | 0.29 | 0.00 | 0.19 | 0.00 |
| hsa-mir-516a-2 | 19q13.42 | 0.29 | 0.00 | 0.19 | 0.00 |
| hsa-mir-519a-2 | 19q13.42 | 0.29 | 0.00 | 0.19 | 0.00 |
| hsa-mir-371 | 19q13.42 | 0.29 | 0.00 | 0.19 | 0.00 |
| hsa-mir-372 | 19q13.42 | 0.29 | 0.00 | 0.19 | 0.00 |
| hsa-mir-373 | 19q13.42 | 0.29 | 0.00 | 0.19 | 0.00 |
| hsa-mir-935 | 19q13.42 | 0.29 | 0.00 | 0.19 | 0.00 |
| hsa-mir-648 | 22q11.21 | 0.29 | 0.00 | 0.52 | 0.00 |
| hsa-mir-185 | 22q11.21 | 0.29 | 0.00 | 0.52 | 0.00 |
| hsa-mir-649 | 22q11.21 | 0.29 | 0.00 | 0.52 | 0.00 |
| hsa-mir-301b | 22q11.21 | 0.29 | 0.00 | 0.52 | 0.00 |
| hsa-mir-130b | 22q11.21 | 0.29 | 0.00 | 0.52 | 0.00 |
| hsa-mir-650 | 22q11.21 | 0.29 | 0.00 | 0.52 | 0.00 |
| hsa-let-7a-3 | 22q13.31 | 0.29 | 0.00 | 0.57 | 0.00 |
| hsa-let-7b | 22q13.31 | 0.29 | 0.00 | 0.57 | 0.00 |

**Table J in S1 File. Upregulated genes with frequent corresponding miR deletions in FL-HCC.**

| Gene | Description | # deleted miRs | miRs | RNA fold change |
| --- | --- | --- | --- | --- |
| LRP8 | low density lipoprotein receptor-related protein 8 | 7 | miR-372, mir-519d, miR-520f, miR-520g, miR-522, miR-527, miR-673 | 2.8 |
| BNC2 | basonuclin 2 | 6 | let-7b, miR-372, miR-519d, miR-520f, miR-520g, miR-527 | 4.4 |
| TRPS1 | trichorhinophalangeal syndrome I | 6 | miR-372, miR-519d, miR-520f, miR-520g, miR-527, miR-935 | 2.7 |
| IGF2BP1 | insulin-like growth factor 2 mRNA binding protein 1 | 5 | let-7b, miR-372, miR-519d, miR-520f, miR-520g | 96.3 |
| DCX | doublecortin | 5 | let-7b, miR-372, miR-498, miR-526b, miR-527 | 9.2 |
| TANC2 | tetratricopeptide repeat, ankyrin repeat and coiled-coil containing 2 | 5 | miR-372, miR-519d, miR-522, miR-526b, miR-527 | 9.2 |
| NOVA1 | neuro-oncological ventral antigen 1 | 5 | let-7b, miR-498, miR-520f, miR-526b, miR-527 | 6.4 |
| DLGAP2 | discs, large (Drosophila) homolog-associated protein 2 | 5 | let-7b, miR-498, miR-520f, miR-526b, miR-527 | 5.5 |
| LIF | leukemia inhibitory factor (cholinergic differentiation factor) | 5 | miR-372, miR-519d, miR-520f, miR-520g, miR-527 | 4.2 |
| PKIA | protein kinase (cAMP-dependent, catalytic) inhibitor alpha | 5 | let-7b, miR-519d, miR-520f, miR-520g, miR-935 | 3.6 |
| TNRC6C | trinucleotide repeat containing 6C | 5 | miR-372, miR-519d, miR-520f, miR-522, miR-527 | 3.2 |
| PLEKHA8 | pleckstrin homology domain containing, family A | 5 | let-7b, miR-519d, miR-520g, miR-527, miR-529e | 2.5 |
| FMNL3 | formin-like 3 | 5 | miR-372, miR-519d, miR-519e, miR-520f, miR-520g | 2.2 |
| MYCN | v-myc myelocytomatosis viral related oncogene | 5 | let-7b, miR-372, miR-519d, miR-520g, miR-527 | 2.1 |
| LRP8 | low density lipoprotein receptor-related protein 8 | 7 | miR-372, mir-519d, miR-520f, miR-520g, miR-522, miR-527, miR-673 | 2.8 |
